# Clinical Factors Associated with Brucella-Related Epididymo-Orchitis: A Retrospective Cohort Study

**DOI:** 10.3390/jcm15114210

**Published:** 2026-05-29

**Authors:** Yakup Gezer, Muhammet Rıdvan Tayşi

**Affiliations:** Infectious Diseases and Clinical Microbiology Clinic, University of Health Sciences, Konya City Hospital, Konya 42020, Türkiye; taysiridvan@gmail.com

**Keywords:** brucellosis, C-reactive protein, endemic region, epididymo-orchitis, genitourinary complications, inflammatory markers, clinical characteristics

## Abstract

**Background/Objectives**: Brucella-related epididymo-orchitis (BEO) is one of the most common genitourinary complications of brucellosis. This study aimed to evaluate the clinical and epidemiological characteristics of BEO in male patients with brucellosis and to identify clinical factors associated with its development. **Methods**: In this retrospective single-center cohort study, adult male patients diagnosed with brucellosis were included. Patients with and without BEO were compared in terms of epidemiological, clinical, and laboratory characteristics. Multivariate logistic regression analysis was performed to identify clinical factors associated with BEO. **Results**: Among 262 male patients with brucellosis, BEO was identified in 44 (16.8%). Of these patients, 38 (86.4%) presented with local scrotal symptoms, and combined involvement of the epididymis and testis was observed in 30 patients (68.2%). In univariable analysis, the patients who developed BEO were younger (33 vs. 44 years, *p* < 0.001), more frequently presented with fever (59.1% vs. 31.2%, *p* < 0.001), and had a shorter symptom duration (2 vs. 4 weeks, *p* < 0.001). In addition, leukocyte counts and C-reactive protein levels were significantly higher in this group (*p* = 0.009 and *p* < 0.001, respectively). In multivariable analysis, younger age (OR: 0.959, 95% CI: 0.930–0.988, *p* = 0.006), shorter symptom duration (OR: 0.852, 95% CI: 0.745–0.975, *p* = 0.020), and the presence of fever (OR: 2.265, 95% CI: 1.103–4.652, *p* = 0.026) were independently associated with BEO. **Conclusions**: BEO is a clinically significant genitourinary complication in male patients with brucellosis, notably associated with younger age, shorter symptom duration, and a more pronounced inflammatory response. In endemic regions, BEO should be considered in the differential diagnosis of patients presenting with fever and acute scrotal symptoms.

## 1. Introduction

Brucellosis is a zoonotic infection that is commonly observed, particularly in the Mediterranean basin, the Middle East, and other endemic regions. The disease is generally transmitted through contact with infected animals or the consumption of unpasteurized dairy products and predominantly affects individuals living in rural areas. Clinical manifestations are often nonspecific and may include fever, fatigue, myalgia, arthralgia, and sweating. Due to its systemic nature, brucellosis may involve multiple organ systems, particularly reticuloendothelial organs [1,2,3].

Brucella species are intracellular pathogens capable of evading host immune responses and disseminating hematogenously to multiple organ systems. This dissemination may result in a wide spectrum of focal complications [4,5,6]. Among these, genitourinary involvement is one of the most common after osteoarticular disease, and epididymo-orchitis represents its most frequent clinical manifestation [3,7]. Approximately one in ten male patients with brucellosis develops epididymo-orchitis [8].

Brucella-related epididymo-orchitis (BEO) is generally characterized by scrotal pain, swelling, and erythema and is most often acute in onset [9,10,11]. Because its clinical presentation closely resembles that of acute bacterial epididymo-orchitis, delays in diagnosis may occur. In addition, BEO may be clinically indistinguishable from other scrotal conditions including testicular tumors and testicular tuberculosis [12,13,14]. Although scrotal ultrasonography (USG) and color Doppler examinations play an important role in diagnosis, imaging findings are often nonspecific and may not reliably differentiate BEO from other inflammatory scrotal diseases [14,15]. Delayed diagnosis and initiation of treatment may lead to serious complications, including testicular abscess, atrophy, and infertility [16,17]. Therefore, early recognition of BEO is critical for timely initiation of appropriate therapy and prevention of adverse outcomes. Furthermore, identifying the clinical characteristics associated with BEO in male patients with brucellosis may facilitate the recognition of genitourinary involvement.

Although several studies have described the clinical features of BEO, data regarding the clinical factors and inflammatory response patterns associated with BEO remain limited. In addition, most available studies are based on small patient series, and comparative evaluations between brucellosis patients with and without BEO are scarce. In this study, we aimed to evaluate the clinical and epidemiological characteristics of BEO in male patients with brucellosis and to identify clinical factors associated with BEO.

## 2. Materials and Methods

### 2.1. Patients

Male patients aged ≥18 years with a first diagnosis of brucellosis were included in the study, whereas female patients were excluded.

The diagnosis of brucellosis was established by detecting a serum agglutination test titer of ≥1/160 in the presence of compatible clinical signs and symptoms. Symptom duration was defined as the time from the onset of clinical signs and symptoms related to brucellosis to the time of diagnosis. The patients were classified according to disease duration as acute (<8 weeks), subacute (8–52 weeks), and chronic (>52 weeks).

The diagnosis of BEO was established in cases with confirmed brucellosis by the presence of clinical findings such as scrotal pain, tenderness, swelling, and erythema, along with ultrasonographic findings consistent with inflammatory changes in the epididymis and/or testis on scrotal USG. Patients without compatible clinical or ultrasonographic findings of epididymo-orchitis were classified as patients without BEO. Osteoarticular involvement was defined as the presence of clinical signs of inflammation such as pain, swelling, increased warmth, erythema, and/or functional limitation in any bone or joint region in patients diagnosed with brucellosis, along with radiological confirmation of these findings.

### 2.2. Study Design

This retrospective single-center observational cohort study was conducted at a tertiary care center in Türkiye between January 2021 and December 2025. Adult male patients with brucellosis with and without BEO were compared according to demographic, epidemiological, clinical, laboratory, and radiological characteristics.

Data were obtained from electronic medical records and included age, duration of symptoms, epidemiological exposures, comorbidities, clinical symptoms and physical examination findings, laboratory parameters, radiological findings, treatment characteristics, and recovery. Epidemiological exposures included living in rural areas, animal contact, and consumption of unpasteurized milk and dairy products. Comorbidities included hypertension, diabetes mellitus, coronary artery disease, malignancy, chronic kidney disease, cerebrovascular disease, and chronic pulmonary disease; the presence of at least one of these conditions was defined as comorbidity. Clinical symptoms and physical examination findings included myalgia, joint pain, fever, tremor, fatigue, weight loss, vomiting, abdominal pain, headache, dysuria, cough, lymphadenopathy, splenomegaly, and hepatomegaly. Laboratory parameters included white blood cell (WBC) count, neutrophil count, monocyte count, lymphocyte count, hemoglobin level, platelet count, alanine aminotransferase (ALT), aspartate aminotransferase (AST), creatinine, C-reactive protein (CRP), and erythrocyte sedimentation rate (ESR). Radiological evaluation included findings from scrotal USG, magnetic resonance imaging, and computed tomography. In all patients with BEO, treatment response was evaluated by the physician who initiated the therapy at the end of the planned treatment duration. Recovery was defined as the complete resolution of all symptoms and findings related to BEO.

This study was designed and reported in accordance with the STROBE guidelines to ensure transparency and completeness in reporting observational research.

### 2.3. Outcomes of the Study

The primary outcome of the study was the presence of BEO and the identification of clinical factors associated with its development among male patients with brucellosis.

Secondary outcomes included the clinical presentation, laboratory and inflammatory parameters, ultrasonographic findings, treatment characteristics, and recovery of patients with BEO.

### 2.4. Statistical Analysis

The normality of continuous variables was assessed using the Shapiro–Wilk test. Non-normally distributed variables were expressed as median and interquartile range (IQR, 25–75%), and categorical variables as number and percentage (%). Univariate analyses were performed to compare patients with and without BEO. Categorical variables were analyzed using the Pearson chi-square or Fisher’s exact test, and continuous variables using the Mann–Whitney U test.

Multivariate logistic regression analysis was performed to identify clinical factors associated with BEO, with variables selected based on clinical relevance and prior evidence from the literature, including age, symptom duration, presence of fever, and underlying comorbidities. Adjusted odds ratios (OR), 95% confidence intervals (CI), and *p*-values were reported for each variable. Model fit was assessed using the Hosmer–Lemeshow goodness-of-fit test (*p* > 0.05). The discriminative ability of the model was evaluated using receiver operating characteristic curve analysis, and the area under the curve (AUC) values were reported.

Given the retrospective design, no a priori power calculation was performed; all eligible patients during the study period were included. The multivariate model included four predictor variables, resulting in an events per variable ratio of 11.

The analysis was performed using Statistical Package for the Social Sciences, version 26.0 (SPSS, IBM Corp., Armonk, NY, USA). A *p*-value < 0.05 was considered statistically significant.

## 3. Results

**Figure 1:**
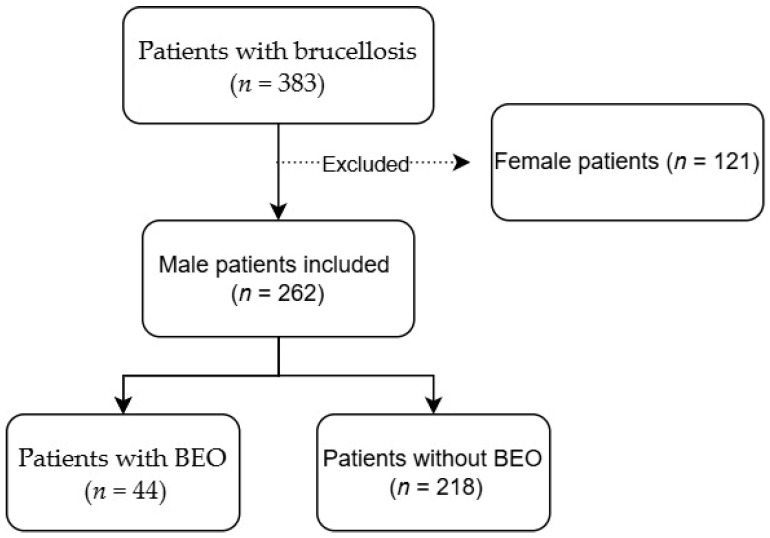
The flow chart of the study; BEO, Brucella-related epididymo-orchitis.

A total of 383 patients with brucellosis were evaluated during the study period. After exclusion of female patients, 262 male patients were included in the final analysis. (Figure 1). The median age was 42 years. Most patients had a history of living in rural areas (69.9%), animal contact (54.2%), or consumption of unpasteurized dairy products (45.4%). The most common symptoms were myalgia (77.1%), joint pain (71.4%), and fever (35.9%). The median symptom duration was 4 weeks (2–6 weeks), and 75.2% of patients were classified as having acute brucellosis (Table 1).

Osteoarticular involvement was observed in 32.1% of the patients. BEO was identified in 44 patients (16.8%). All the patients with BEO presented with scrotal pain, while scrotal erythema and scrotal swelling were observed in 86.4% and 77.3% of the cases, respectively. Thirty-eight patients (86.4%) initially presented directly to the urology outpatient clinic with these complaints and were thereafter referred to the infectious diseases department for brucellosis treatment after being diagnosed with BEO. Detailed history-taking revealed that systemic brucellosis symptoms had preceded scrotal manifestations in these patients. The remaining six patients (13.6%) initially presented to the infectious diseases outpatient clinic with non-scrotal manifestations of brucellosis. Scrotal symptoms developed during the course of the disease after initiation of anti-brucellosis therapy, and these patients were subsequently referred to the urology department for further evaluation and management. Among patients with BEO, the median interval between the onset of initial symptoms and the development of scrotal manifestations was 2 weeks (1–4 weeks).

Scrotal USG and color Doppler imaging were performed in all the patients diagnosed with BEO. Combined epididymal and testicular involvement was identified in 30 patients (68.2%), including unilateral epididymo-orchitis in 26 patients and bilateral epididymo-orchitis in four patients. Among the remaining patients, nine (20.4%) had unilateral orchitis and five (11.4%) had unilateral epididymitis. Ultrasonography demonstrated enlargement and heterogeneous echotexture in all affected structures. Color Doppler imaging revealed increased vascularity in the epididymis and/or testis in all cases. In addition, reactive hydrocele was detected in five patients (11.4%), while testicular abscess was observed in one patient (2.3%).

Treatment regimens for patients with BEO were determined based on the presence of concomitant osteoarticular involvement. In all patients without osteoarticular involvement, including epididymo-orchitis, one of the combinations of doxycycline (200 mg/day, oral) plus either rifampicin (600 mg/day, oral) or streptomycin (1 g/day, 14–21 days, intramuscular) was administered as treatment for 6–8 weeks. In the patients with osteoarticular involvement, regardless of the presence of epididymo-orchitis, the combination of oral doxycycline and rifampicin was administered for at least 3 months, with additional intramuscular streptomycin given for 14–21 days. Of the 44 patients with BEO, three had concomitant osteoarticular involvement and received prolonged doxycycline-rifampicin therapy combined with streptomycin. Among the remaining patients, 35 patients received doxycycline plus rifampicin, whereas six patients received doxycycline plus streptomycin. No patient required surgical intervention. At the end of treatment, recovery was observed in all the patients with BEO.

Comparative analysis between the patients with and without BEO demonstrated that the patients in the BEO group were younger (33 vs. 44 years, *p* < 0.001), had a shorter symptom duration (2 vs. 4 weeks, *p* < 0.001), and more frequently presented with fever (59.1% vs. 31.2%, *p* < 0.001). Osteoarticular involvement was significantly less common in the BEO group (6.8% vs. 37.2%, *p* < 0.001). The presence of comorbidity was similar between the groups (6.8% vs. 15.6%, *p* = 0.127). Although no statistically significant difference was detected in the distribution of clinical stages, the acute phase was observed at a higher rate in the BEO group (88.6% vs. 72.5%, *p* = 0.06) (Table 1). In the patients with BEO, WBC count (8.7 vs. 7.6 (10^9^/L); *p* = 0.009) and CRP (35.1 vs. 5 (mg/L); *p* < 0.001) levels were found to be significantly higher (Table 2).

Multivariate logistic regression analysis identified younger age (OR: 0.959, 95% CI: 0.930–0.988, *p* = 0.006), shorter symptom duration (OR: 0.852, 95% CI: 0.745–0.975, *p* = 0.020), and the presence of fever (OR: 2.265, 95% CI: 1.103–4.652, *p* = 0.026) as clinical factors associated with BEO (Table 3). The model demonstrated good discriminative ability (AUC = 0.781, 95% CI: 0.717–0.846; *p* < 0.001) and acceptable calibration (Hosmer–Lemeshow *p* = 0.287).

## 4. Discussion

In this study, the frequency of epididymo-orchitis among the male patients with brucellosis was 16.8%. Approximately three-quarters of the patients had a history of rural residence, while nearly half reported animal contact or consumption of unpasteurized dairy products. Multivariate analysis identified younger age, shorter symptom duration, and the presence of fever as clinical factors independently associated with BEO.

BEO represents the most common form of genitourinary involvement in brucellosis [9]. In male patients, its reported incidence ranges between 2.5% and 18.8% in the literature [8,18,19,20,21,22]. In our cohort from an endemic region of Türkiye, the observed rate of BEO (16.8%) was consistent with previously published data.

Türkiye remains an endemic country for brucellosis, particularly in rural areas where close contact with livestock and consumption of unpasteurized dairy products are still common [3,9,23]. Consistent with this epidemiological background, approximately three-quarters of our patients had a history of rural residence, while nearly half reported animal contact or consumption of unpasteurized dairy products. These findings are in line with previous reports identifying traditional exposure patterns as major risk factors for brucellosis transmission in endemic regions [10,12,24,25,26].

Consistent with previous reports, all the patients with BEO in this study presented with scrotal pain, while scrotal erythema and edema were observed in the majority of cases [17,19,27]. In a study from Iran including 50 patients with BEO, all patients reportedly presented to healthcare facilities with scrotal symptoms [28]. Similarly, in our cohort, most patients were admitted to the urology outpatient clinic with these complaints. These findings suggest that BEO more commonly presents with local scrotal manifestations rather than systemic symptoms of brucellosis. However, these local findings are nonspecific and may also be observed in acute bacterial epididymo-orchitis. Nevertheless, the management of BEO requires prolonged combination anti-brucella therapy, whereas acute bacterial epididymo-orchitis is typically treated with shorter, pathogen-directed antibiotic regimens, making this distinction clinically important [12,13,14,28,29]. Alarbid et al. reported that in endemic regions, epidemiological exposure history, relative lymphocytosis, and relative neutropenia may help differentiate BEO from nonspecific bacterial epididymo-orchitis [12]. However, this differential assessment could not be performed in our study due to its scope, and since all included patients had a confirmed diagnosis of brucellosis.

In our series, all the patients with BEO demonstrated findings consistent with inflammation on scrotal ultrasonography and color Doppler imaging. The most common pattern was combined epididymal and testicular involvement, and all cases showed enlargement, heterogeneous echotexture, and increased vascularity in the affected structures. These findings are consistent with the most frequently reported ultrasonographic features of inflammation in BEO in the literature [14,17,20,26]. However, since these imaging features may also be observed in acute bacterial epididymo-orchitis, scrotal ultrasonography and Doppler imaging alone may not be sufficient for differential diagnosis [15,30]. Therefore, ultrasonographic evaluation should be interpreted in conjunction with clinical findings and epidemiological history.

In our study, the patients who developed BEO were significantly younger, a finding that is consistent with previous reports indicating that BEO predominantly occurs in younger male patients [10,17,19,31]. Given that testicular inflammation may have adverse effects on spermatogenesis and may potentially reduce fertility in the long term, the occurrence of BEO in men of reproductive age is of particular clinical importance [10,16,17,32]. In addition, the shorter symptom duration observed in patients with BEO suggests that epididymo-orchitis may represent one of the early clinical manifestations of brucellosis [17,19,27]. In contrast, osteoarticular involvement has been reported to be associated with a longer symptom duration and more advanced stages of disease [33]. The lower frequency of osteoarticular involvement in the BEO group in our study is also consistent with this observation.

Fever was more frequently observed in the patients with BEO, and WBC and CRP levels were significantly higher in this group. In the multivariate analysis, fever was identified as an independent factor associated with BEO. These findings are consistent with previous reports demonstrating elevated inflammatory markers in BEO [17,19,25,26]. This may be explained by the dissemination of Brucella spp. to the testicular tissue via infected cells, resulting in disruption of the blood–testis barrier and increased local cytokine release [4]. In contrast, elevated inflammatory markers are not always prominent in patients with osteoarticular involvement, as reported in previous studies [33,34]. Taken together, these findings suggest that in male patients diagnosed with brucellosis, genitourinary involvement should be considered, particularly in younger patients and those presenting with a shorter symptom duration, fever, leukocytosis, and elevated CRP levels.

This study has several limitations. First, the retrospective single-center design may limit the generalizability of the findings, and incomplete data capture with variability in data standardization cannot be excluded. Second, the possibility of detection bias should be acknowledged, since scrotal ultrasonography and urological assessment were more likely to be performed in symptomatic patients. Therefore, mild or subclinical cases of BEO may have remained undetected. In addition, BEO-specific clinical severity, symptom duration, and prognostic parameters could not be systematically assessed across all patients. Furthermore, the absence of long-term follow-up data restricts the interpretation of long-term outcomes of BEO. Residual confounding may also be present despite the use of multivariable analysis, as the regression model included a limited number of variables and unmeasured clinical factors may still have influenced the observed associations. Prospective multicenter studies are needed to better define the clinical characteristics and long-term urogenital outcomes of BEO.

A major strength of this study is the analysis of the clinical, epidemiological, ultrasonographic, and laboratory characteristics of BEO in a male brucellosis cohort from an endemic region. Moreover, by providing a comparative analysis between brucellosis patients with and without BEO, this study contributes to the limited existing literature on the clinical and inflammatory features associated with BEO.

## 5. Conclusions

BEO is a clinically significant genitourinary complication in male patients with brucellosis, notably associated with younger age, shorter symptom duration, and a more pronounced inflammatory response. In endemic regions, a thorough epidemiological history should be obtained and BEO should be considered in the differential diagnosis of patients presenting with fever and acute scrotal symptoms.

## Figures and Tables

**Table 1:** Demographic, epidemiological, and clinical characteristics of male brucellosis patients with and without epididymo-orchitis.

Variables	All Patients (*n* = 262)	Patients with BEO (*n* = 44)	Patients Without BEO (*n* = 218)	*p* Value
Age, years	42 (31–54)	33 (23.3–43.5)	44 (33–56)	<0.001
Presence of comorbidity	37 (14.1)	3 (6.8)	34 (15.6)	0.127
Hypertension	18 (6.9)	1 (2.3)	17 (7.8)	0.324
Diabetes mellitus	14 (5.3)	0	14 (6.4)	0.136
Coronary artery disease	13 (5)	1 (2.3)	12 (5.5)	0.702
Chronic kidney disease	2 (0.8)	1 (2.3)	1 (0.5)	0.308
Malignancy	2 (0.8)	0	2 (0.9)	>0.999
Other	5 (1.9)	0	5 (2.3)	0.040
History of living in rural areas	183 (69.9)	35 (79.6)	148 (67.9)	0.124
Animal contact	142 (54.2)	23 (52.3)	119 (54.6)	0.779
History of consumption of unpasteurized milk and dairy products	123 (45.4)	24 (54.6)	99 (45.4)	0.272
Symptoms				
Myalgia	202 (77.1)	39 (88.6)	163 (74.8)	0.046
Joint pain	187 (71.4)	27 (61.4)	160 (73.4)	0.108
Fever	94 (35.9)	26 (59.1)	68 (31.2)	<0.001
Tremor	66 (25.2)	15 (34.1)	51 (23.4)	0.136
Fatigue	48 (18.3)	9 (20.5)	39 (17.9)	0.688
Weight loss	37 (14.1)	6 (13.6)	31 (14.2)	0.920
Vomiting	31 (11.8)	5 (11.4)	26 (11.9)	0.916
Abdominal pain	19 (7.3)	11 (25)	8 (3.7)	<0.001
Headache	15 (5.7)	5 (11.4)	10 (4.6)	0.144
Dysuria	10 (3.8)	8 (18.2)	2 (0.9)	<0.001
Cough	1 (0.4)	1 (2.3)	0	0.168
Clinical stage				0.06
Acute brucellosis	197 (75.2)	39 (88.6)	158 (72.5)	
Subacute brucellosis	55 (21)	5 (11.4)	50 (22.9)	
Chronic brucellosis	10 (3.8)	0	10 (4.6)	
Symptom duration, weeks	4 (2–6)	2 (1–4)	4 (2–9)	<0.001
Focal and systemic organ involvement				
Lymphadenopathy	28 (10.7)	4 (9.1)	24 (11)	0.707
Hepatomegaly	32 (12.2)	6 (13.6)	26 (11.9)	0.752
Splenomegaly	27 (10.3)	5 (11.4)	22 (10.1)	0.800
Osteoarticular involvement	84 (32.1)	3 (6.8)	81 (37.2)	<0.001

BEO: Brucella-related epididymo-orchitis; IQR: interquartile range. Categorical variables are presented as *n* (%); numerical variables are expressed as median (25th and 75th IQR).

**Table 2:** Laboratory findings of male brucellosis patients with and without epididymo-orchitis.

Variables	All Patients (*n* = 262)	Patients with BEO (*n* = 44)	Patients Without BEO (*n* = 218)	*p* Value
White blood cell count, 10^9^/L	7.7 (6.6–9.8)	8.7 (6.9–11.5)	7.6 (6.5–9.5)	0.009
Neutrophil count, 10^9^/L	4.4 (3.5–5.9)	4.7 (3.7–7.7)	4.3 (3.5–5.6)	0.051
Lymphocyte count, 10^9^/L	2.4 (1.9–2.9)	2,5 (2–3.2)	2.4 (1.9–2.9)	0.250
Monocyte count, 10^9^/L	0.7 (0.5–0.8)	0.7 (0.5–1)	0.6 (0.5–0.8)	0.147
Hemoglobin count, g/dL	15 (14–15.9)	14.5 (13.7–15.3)	15.2 (14–16)	0.023
Platelet count, 10^9^/L	255.5 (209–304.3)	256 (207.5–305.8)	255.5 (209–303.3)	0.810
AST, U/L	24 (19–32.3)	27 (18.3–34.5)	23 (19–32)	0.304
ALT, U/L	24 (17–38.3)	28 (19.3–51.8)	23 (17–35)	0.042
Creatinine, mg/dL	0.8 (0.7–0.9)	0,8 (0.7–0.9)	0.8 (0.7–0.9)	0.799
CRP, mg/L	7.3 (1.7–30.5)	35.1 (14.5–84.3)	5 (1.5–20)	<0.001
ESR, mm/h	12.5 (5–29)	13.5 (6–30.5)	12 (5–29)	0.555

CRP: C-reactive protein; ESR: erythrocyte sedimentation rate; AST: aspartate aminotransferase; ALT: alanine aminotransferase; BEO: Brucella-related epididymo-orchitis; IQR: interquartile range. Numerical variables are expressed as median (25th and 75th IQR).

**Table 3:** Multivariate logistic regression analysis of clinical factors associated with epididymo-orchitis in male brucellosis patients.

Variables	Univariate Analysis		Multivariate Analysis	
	OR (95%CI)	*p* Value	OR (95%CI)	*p* Value
Age, years	0.949 (0.924–0.974)	<0.001	0.959 (0.930–0.988)	0.006
Symptom duration, weeks	0.799 (0.694–0.921)	0.002	0.852 (0.745–0.975)	0.020
Fever	3.186 (1.637–6.201)	0.001	2.265 (1.103–4.652)	0.026
Presence of comorbidity	0.396 (0.116–1.352)	0.139	0.977 (0.237–4.031)	0.974

OR: odds ratio, CI: confidence interval.

## Data Availability

The datasets generated and analyzed during the current study are available from the corresponding author upon reasonable request.

## Abbreviations

IQR	interquartile range
AUC	area under the receiver operating characteristic curve
OR	odds ratio
CI	confidence interval
BEO	brucella-related epididymo-orchitis
WBC	white blood cell
CRP	C-reactive protein
ESR	erythrocyte sedimentation rate
AST	aspartate aminotransferase
ALT	alanine aminotransferase
USG	ultrasonography
